# The Classification of Autosomal Recessive Cerebellar Ataxias: a Consensus Statement from the Society for Research on the Cerebellum and Ataxias Task Force

**DOI:** 10.1007/s12311-019-01052-2

**Published:** 2019-07-02

**Authors:** Marie Beaudin, Antoni Matilla-Dueñas, Bing-Weng Soong, Jose Luiz Pedroso, Orlando G. Barsottini, Hiroshi Mitoma, Shoji Tsuji, Jeremy D. Schmahmann, Mario Manto, Guy A Rouleau, Christopher Klein, Nicolas Dupre

**Affiliations:** 1 Axe Neurosciences, CHU de Québec–Université Laval, Québec, QC Canada; 2 Department of Medicine, Faculty of Medicine, Université Laval, Quebec City, QC Canada; 3 Department of Neuroscience, Health Sciences Research Institute Germans Trias i Pujol (IGTP), Universitat Autònoma de Barcelona, Badalona, Barcelona Spain; 4 Department of Neurology, Shuang Ho Hospital and Taipei Neuroscience Institute, Taipei Medical University, Taipei, Taiwan Republic of China; 5 National Yang-Ming University School of Medicine, Taipei Veterans General Hospital, Taipei, Taiwan Republic of China; 6 Ataxia Unit, Department of Neurology, Universidade Federal de São Paulo, São Paulo, SP Brazil; 7 Medical Education Promotion Center, Tokyo Medical University, Tokyo, Japan; 8 The University of Tokyo, Tokyo, Japan; 9 International University of Health and Welfare, Chiba, Japan; 10 Department of Neurology, Massachusetts General Hospital and Harvard Medical School, Boston, MA USA; 11 Service de Neurologie, Médiathèque Jean Jacquy, CHU-Charleroi, 6000 Charleroi, Belgium; 12 Service des Neurosciences, UMons, Mons, Belgium; 13 McGill University, Montreal, QC Canada; 14 Mayo Clinic, Rochester, MN USA

**Keywords:** Spinocerebellar degenerations, Cerebellar ataxia, Friedreich ataxia, Ataxia telangiectasia, Genetics, Classification

## Abstract

There is currently no accepted classification of autosomal recessive cerebellar ataxias, a group of disorders characterized by important genetic heterogeneity and complex phenotypes. The objective of this task force was to build a consensus on the classification of autosomal recessive ataxias in order to develop a general approach to a patient presenting with ataxia, organize disorders according to clinical presentation, and define this field of research by identifying common pathogenic molecular mechanisms in these disorders. The work of this task force was based on a previously published systematic scoping review of the literature that identified autosomal recessive disorders characterized primarily by cerebellar motor dysfunction and cerebellar degeneration. The task force regrouped 12 international ataxia experts who decided on general orientation and specific issues. We identified 59 disorders that are classified as primary autosomal recessive cerebellar ataxias. For each of these disorders, we present geographical and ethnical specificities along with distinctive clinical and imagery features. These primary recessive ataxias were organized in a clinical and a pathophysiological classification, and we present a general clinical approach to the patient presenting with ataxia. We also identified a list of 48 complex multisystem disorders that are associated with ataxia and should be included in the differential diagnosis of autosomal recessive ataxias. This classification is the result of a consensus among a panel of international experts, and it promotes a unified understanding of autosomal recessive cerebellar disorders for clinicians and researchers.

## Introduction

The classification of hereditary ataxias represents a significant challenge due to the large number of neurological and metabolic diseases that present with cerebellar dysfunction and the phenotypic heterogeneity in known genetically defined disorders. Indeed, ataxia is a presenting feature in degenerative disorders that target mainly the cerebellum, but it may be present in hereditary spastic paraplegias, inborn errors of metabolism, and various encephalopathies. Proper classification and phenotypic understanding is of primary importance in this field where the high prevalence of repeat expansion disorders, which are not adequately covered by the next-generation sequencing (NGS) techniques [1, 2], precludes NGS as a first diagnostic step and requires phenotypic evaluation to perform custom gene testing when applicable. Nevertheless, autosomal recessive cerebellar ataxias have remained an ill-defined and disorganized group of disorders for two main reasons. First, unlike the dominant ataxias that have been organized with a numerical naming system, recessive disorders presenting with ataxia have been named in a highly heterogeneous manner according to clinical features, physicians’ surname, or regions of high prevalence. Second, several recessive multisystemic or complex metabolic disorders present with ataxia, such that it is difficult to properly circumscribe this group of disorders and classify it in a meaningful way for both clinicians and researchers. Hence, the Society for Research on the Cerebellum and Ataxias (SRCA) Task Force on the Classification of Recessive Cerebellar Ataxias was created in 2016 to regroup a panel of international ataxia experts in order to propose a classification relevant to clinical practice and researchers. As a first step, we undertook a systematic scoping review of the literature to identify all recessive disorders presenting with ataxia, select those in which cerebellar degeneration was a core feature, and propose a first classification. This systematic scoping review has been previously published [3] and served as the basis for the current work.

Recently, the Movement Disorder Society Task Force on Classification and Nomenclature of Genetic Movement Disorders proposed a revised naming system based on the gene name associated with a phenotypical prefix. They presented a list of 92 gene-defined recessive disorders associated with ataxia for which this naming system would be applied and an exhaustive list of disorders that may occasionally present with ataxia [4]. This represents a useful reference for interpretation of NGS results. However, in a significant number of listed disorders, the cerebellum is only one of the many affected organs in multisystemic and metabolic disorders. For example, maple syrup urine disease, caused by BCKDHB mutations, and congenital disorders of glycosylation 1a, 1c, and 1q have been included. These disorders are inborn errors of metabolism characterized by developmental delay, hypotonia, and metabolic defects, and ataxia is only mild, found in a minority of patients, or present solely during episodes of metabolic decompensation. Hence, there remains a need for a classification system that focuses on disorders affecting primarily the cerebellum and organizes clinical and paraclinical information to promote an understanding of cerebellar disorders useful not only to ataxia experts but also to general neurologists, learners, patients, and researchers.

The objective of this task force was to build a consensus on the classification of autosomal recessive ataxias in order to develop a general approach to a patient presenting with ataxia, organize disorders according to clinical presentation, and define this field of research by identifying common pathophysiological mechanisms in recessive disorders presenting with ataxia. This aims at bringing together clinicians and researchers to promote a common understanding of recessive cerebellar disorders in order to advance research and improve patient care.

## Materials and Methods

The first step was to identify all recessive disorders presenting with ataxia. Recessive cerebellar ataxias were defined as disorders with autosomal recessive inheritance characterized by a cerebellar motor syndrome of gait ataxia, dysmetria, adiadochokinesia, nystagmus, and dysarthria associated with cerebellar degeneration as demonstrated by imagery or pathology. A pathogenic mutation had to be identified in at least two independent families for a specific gene to be included. Purely malformative disorders were excluded, and disorders with complex phenotypes where ataxia is a secondary or late feature were also excluded. We conducted a systematic scoping review of the literature to identify relevant reports. The methodology and results of this systematic review have been published previously [3]. In the first publication, this review process had allowed the identification of 2354 records and was current as of September 2016. The literature search was updated and is current as of October 2018.

The second step was to regroup a panel of 12 international ataxia experts to create a logical classification system and build a consensus. Ataxia experts were identified from various geographical regions and areas of expertise within the field of ataxias, ensuring proper representation of regional differences in prevalence and clinical approach to ataxias. Discussions spanned over 2 years, included meetings at two SRCA international conferences, and concerned general orientation, clinical approach, specific disorders, classification issues, and regional specificities. The first author (MB) reviewed identified records for inclusion, extracted clinical, epidemiological, and molecular data to build the classifications and wrote the text integrating all authors’ input and comments. All authors approved the final manuscript and list of included disorders.

## Results

The final list of included autosomal recessive cerebellar ataxias is presented in Table 1 and includes 59 primary recessive ataxias, which regroup 15 disorders that are more prevalent and widely distributed and 44 disorders that are less frequent and reported only in certain populations or few families. Because ethnic and regional specificities are an essential element to consider in the appraisal of a patient with a recessive ataxia, areas where the disorder has been reported to date are listed. Metabolic or mitochondrial disorders where ataxia is only a secondary nonspecific finding in a multisystemic phenotype were excluded, as cerebellar pathology is not central in these disorders. However, clinicians must bear in mind that some of these disorders may present with a milder juvenile or adult onset phenotype where cerebellar ataxia may predominate, for example, in Niemann-Pick disease type C, Tay-Sachs disease, sialic acid storage disorders, congenital disorders of glycosylation, and Zellweger spectrum disorders. As some of these metabolic disorders may benefit from early treatment, clinicians must keep a high index of suspicion to test for these disorders, and they should be included in large NGS gene panels for ataxia. These and other complex disorders that may occasionally present with ataxia are presented in Table 2. This second list is not exhaustive and presents only the main or most frequent disorders occasionally associated with ataxia. Disorders in which the cerebellar phenotype is not clearly established have been excluded.

**Table 1:** Primary autosomal recessive cerebellar ataxias

MDS nomenclature^1^ or gene name	Alternate nomenclature^2^	OMIM	Geographic specificities	Additional clinical clues and neuroimaging findings	References
Most prevalent ataxias					
ATX-FXN	FRDA	229300	Most prevalent in populations of European descent, Middle East, and North Africa; absent in Far East populations	Bilateral Babinski sign, square-wave jerks, scoliosis, hypertrophic cardiomyopathy, sensory involvement, teenage onset, spinal cord atrophy, absence of cerebellar atrophy	[5, 6]
ATX-ATM	AT	208900	Second most common cause of recessive ataxia worldwide, especially in regions with low inbreeding	Telangiectasias, oculomotor apraxia, photosensitivity, immunodeficiency, predisposition for cancer, dystonia, myoclonus, choreoathetosis, tremor, elevation of α-fetoprotein, infantile onset, cerebellar atrophy	[7–9]
ATX-APTX	AOA1/EAOH	208920	Most prevalent in Japan; second most prevalent ataxia in Portugal	Oculomotor apraxia, cognitive impairment, axonal motor polyneuropathy, late onset of hypoalbuminemia, elevated α-fetoprotein and hypercholesterolemia, childhood onset, cerebellar atrophy	[10–12]
ATX-SETX	AOA2	606002	Worldwide, second most prevalent in Eastern France	Axonal sensorimotor polyneuropathy, pyramidal signs, oculomotor apraxia, head tremor, chorea, dystonia, elevation of α-fetoprotein, teenage onset, cerebellar atrophy	[13–15]
ATX/HSP-SACS	ARSACS	270550	Worldwide	Spastic paraparesis, retinal striation with thickened retinal nerve fibers, sensorimotor neuropathy, pes cavus, infantile or childhood onset, anterior superior cerebellar atrophy, occasional T2-weighted linear hypointensities in pons	[16, 17]
POLG	MIRAS, SANDO, SCAE	607459	Prevalent in populations of European descent, especially Scandinavia, UK, and Belgium	Cerebellar and sensory ataxia, dysarthria, progressive external ophthalmoplegia, myoclonus, epilepsy, myopathy, migraine, variable age at onset, signal abnormalities in the cerebellum and thalamus	[18–20]
ATX-SYNE1	ARCA1	610743	Worldwide	Pure cerebellar ataxia with occasional upper and/or lower motor neuron involvement, cognitive impairment, late onset, cerebellar atrophy	[21–23]
HSP/ATX-SPG7	SPG7	607259	Described worldwide, frequent in Europe	Spasticity, pyramidal signs, optic neuropathy, ptosis, ophthalmoparesis, bladder dysfunction, adult onset, cerebellar atrophy	[24, 25]
COQ8A (ATX-ADCK3)	ARCA2	612016	European descent, Algeria, Middle East	Exercise intolerance, epilepsy, myoclonus, developmental delay, intellectual disability, childhood onset, cerebellar atrophy, occasional stroke-like cerebral lesions	[26, 27]
ATX-ANO10	ARCA3	613728	European descent, Middle East, West Indies, Japan	Pure cerebellar ataxia with occasional upper motor neuron signs, cognitive impairment, epilepsy, nystagmus, teenage or adult onset, cerebellar atrophy	[28–30]
ATX-TTPA	AVED	277460	Worldwide, high prevalence around Mediterranean sea	Dorsal column involvement, areflexia, retinitis pigmentosa, head titubation, low serum vitamin E, skeletal deformities, teenage onset, spinal cord atrophy, occasional cerebellar atrophy	[31–33]
ATX-CYP27A1	CTX	213700	Worldwide	Dementia, pyramidal signs, epilepsy, tendon xanthomas, atherosclerosis, cataracts, diarrhea, elevated serum cholestanol, polyneuropathy, childhood to adult onset, variable cerebellar atrophy, cerebellar or cerebral white matter anomalies	[34–36]
ATX-SIL1	MSS	248800	Worldwide	Cataracts, intellectual disability, myopathy, short stature, childhood onset, cerebellar atrophy	[37, 38]
TWNK (ATX-C10orf2)	IOSCA/MTDPS7	271245	Described worldwide, highly prevalent in Finland	Athetosis, sensory axonal neuropathy, hypotonia, optic atrophy, ophthalmoplegia, sensorineural deafness, epilepsy, hypogonadism, liver involvement, infantile onset, atrophy of the brainstem and cerebellum	[39, 40]
Rare ataxias or described only in few families					
ATX-ABHD12	PHARC	612674	Europe, USA, Middle East, Algeria	Demyelinating sensorimotor neuropathy, pes cavus, cataracts, hearing loss, retinitis pigmentosa, teenage onset, cerebellar atrophy	[41, 42]
ATX/HSP-AFG3L2	SPAX5	614487	Colombia, Saudi Arabia	Ataxia, spasticity, oculomotor apraxia, myoclonic epilepsy, neuropathy, extrapyramidal involvement, optic atrophy, severe cases with developmental regression, microcephaly, hypsarrhythmia and intractable epilepsy, infantile to childhood onset, cerebellar atrophy	[43, 44]
ATCAY	Cayman ataxia	601238	Grand Cayman Islands, Pakistan	Psychomotor retardation, hypotonia, strabismus, bradykinesia, occasional dystonia, neonatal or infantile onset, cerebellar hypoplasia	[45, 46]
ATX-CA8	CAMRQ3	613227	Iran, Saudi Arabia, Syria	Mild intellectual disability, occasional quadrupedal gait, tremor, hyperreflexia, congenital onset, cerebellar atrophy, periventricular white matter anomalies	[47, 48]
HSP/ATX-CAPN1	SPG76	616907	Europe, Middle East, Brazil, Japan, Punjab	Pyramidal signs, pes cavus, dysarthria, ataxia, slow saccades, cognitive impairment, teenage to adult onset, cerebellar vermian atrophy	[49, 50]
HSP/ATX-CLCN2	Leucoencephalopathy with ataxia	615651	Europe, North Africa, Turkey, Japan	Chorioretinopathy, optic neuropathy, learning disability, headaches, occasional mild spasticity, childhood to adult onset, T2 hypersignal in cerebellar and cerebral peduncles with internal capsule, myelin microvacuolation	[51, 52]
COA7	MC4D, SCAN3	220110	Italy, Japan	Sensorimotor neuropathy, hyporeflexia, mild cognitive impairment, elevated serum creatine kinase, elevated lactate and pyruvate, ragged red fibers, infantile to childhood onset, cerebellar atrophy, supratentorial leucopathy, spinal cord atrophy	[53, 54]
ATX-COX20	Mitochondrial complex IV deficiency	220110	Turkey	Growth retardation, pyramidal signs, sensory neuropathy, extrapyramidal features, elevation of blood lactate, childhood or teenage onset, cerebellar atrophy	[55, 56]
ATX-CWF19L1	SCAR17	616127	Turkey, Netherlands	Intellectual disability, congenital to infantile onset, cerebellar atrophy	[57, 58]
HSP/ATX-CYP7B1	SPG5A	270800	Worldwide, prevalent in Europe	Pyramidal signs, dorsal column sensory deficits, urge incontinence or voiding, childhood or teenage onset, white matter lesions	[59, 60]
ATX/HSP-DARS2	LBSL	611105	Worldwide, high carrier rate in Finland	Pyramidal signs, dorsal column dysfunction, axonal neuropathy, tremor, cerebral lactic acidosis, seizures, infantile to adult onset, signal abnormalities in cerebral white matter and specific brainstem and spinal cord tracts	[61, 62]
ATX-DNAJC19	DCMA/MGCA5	610198	Canadian Hutterite population, Finland, Turkey	Dilated cardiomyopathy, nonprogressive cerebellar ataxia, intellectual disability, testicular dysgenesis, anemia, increased urinary 3-methylglutaconic acid, infantile onset, progressive cerebellar atrophy	[63–65]
HSP/ATX-GBA2	SPG46	614409	Tunisia, Cyprus, Italy, Norway	Pyramidal signs, spastic dysarthria, cognitive impairment, hearing loss, cataracts, urge incontinence, axonal sensorimotor neuropathy, childhood onset, cerebellar and cerebral atrophy, thin corpus callosum	[66, 67]
GDAP2	–	–	Belgium, Dutchland, Egypt	Pyramidal signs, cognitive impairment, adult onset, cerebellar atrophy	[68]
ATX/HSP-GJC2	HLD2 or Pelizaeus-Merzbacher-like disease	608804	Worldwide	Nystagmus, hypotonia progressing to spastic tetraparesis, developmental delay, dystonia, chorea, neonatal to infantile onset, diffuse hypomyelination	[69, 70]
MYC/ATX-GOSR2	Progressive myoclonic epilepsy 6	614018	North Sea region	Areflexia, myoclonic seizures, scoliosis, late cognitive impairment, axonal sensory neuropathy and anterior horn cell involvement, raised creatine kinase, infantile onset, occasional cerebellar atrophy	[71, 72]
ATX-GRID2	SCAR18	616204	Middle East, Mexico, Morocco	Tonic upgaze, vertical nystagmus, oculomotor apraxia, intellectual disability, developmental delay, hypotonia, infantile onset, cerebellar atrophy; possible autosomal dominant transmission	[73, 74]
GRM1	SCAR13	614831	Roma ethnic group in Bulgaria	Developmental delay, intellectual disability, occasional pyramidal signs, short stature, seizures, congenital onset, cerebellar atrophy; allelic with SCA44	[75, 76]
ATX-GRN	CLN11	614706	Italy, Portugal, Brazil	Myoclonic epilepsy, retinopathy, dementia, adult onset, cerebellar atrophy	[77, 78]
ATX-ITPR1	Gillespie syndrome	206700	Brazil, Europe, North Africa, Middle East, Asia, Caribbean Islands	Autosomal recessive and dominant transmission. Nonprogressive cerebellar ataxia, iris hypoplasia, hypotonia, intellectual disability, facial dysmorphism, neonatal onset, progressive cerebellar atrophy; allelic with SCA15 and SCA29	[79, 80]
HSP/ATX-KIF1C	SPAX2/SPG58	611302	Palestine, Morocco, Turkey, Germany	Spastic paraparesis with pyramidal signs, tremor, childhood or teenage onset, T2 hyperintensity in internal capsules, parietal and occipital white matter, cerebellar peduncles, and pyramidal tracts	[81, 82]
ATX-KCNJ10	EAST/SeSAME syndrome	612780	Africa, Middle East, India, Caucasian, Afro-Caribbean population	Epilepsy, sensorineural deafness, intellectual disability, tubulopathy and electrolyte imbalance, hypotonia progressing to spasticity, infantile onset, cerebellar hypoplasia, signal anomaly in dentate nuclei	[83–85]
ATX-L2HGDH	L-2-hydroxyglutaric aciduria	236792	Worldwide	Developmental delay, macrocephaly, hypotonia, elevated levels of L-2-hydroxyglutaric acid, infantile to adult onset, subcortical white matter, dentate nucleus and basal ganglia signal anomalies, cerebellar atrophy	[86, 87]
ATX-MRE11A	ATLD	604391	Described in Europe, Saudi Arabia, Canada, Pakistan and Japan	Oculomotor apraxia, extrapyramidal movement disorders, occasional myoclonus, childhood onset, cerebellar atrophy	[88, 89]
MTPAP	SPAX4	613672	Amish families	Pyramidal signs, optic atrophy, sensibility to ionizing radiations, developmental delay, cognitive impairment, growth failure, infantile onset	[90, 91]
ATX-PEX10	PBD 6B or ZSD	614871	Caucasians, Japan	Axonal motor or sensorimotor neuropathy, variable cognitive impairment, nystagmus, hypo or hyperreflexia, childhood to adolescent onset, cerebellar atrophy	[92, 93]
ATX-PMPCA	SCAR2	213200	Lebanon, France, French Canadians	Intellectual disability, hypotonia, short stature, severe phenotype with lactic academia and ophthalmoplegia, congenital or infantile onset, cerebellar atrophy	[94–96]
PNKP	AOA4	616267	European descent	Dystonia, oculomotor apraxia, sensorimotor polyneuropathy, cognitive impairment, childhood onset, cerebellar atrophy	[97–99]
ATX/HSP-PNPLA6	BNS/GHS/OMCS	215470, 275400	Worldwide	Hypogonadotropic hypogonadism, chorioretinal dystrophy, pyramidal signs, childhood onset, atrophy of the cerebellum and pons; allelic to HSP39	[100, 101]
ATX/HSP-POLR3A	HLD7, 4H syndrome	607694	Worldwide	Tremor, variable cognitive impairment, spasticity, hyperreflexia, variable hypodontia and dysmorphism, hypogonadotropic hypogonadism, myopia, short stature, infantile to childhood onset, diffuse cerebral hypomyelination, cerebellar atrophy, thin corpus callosum, T2 hypointense thalamus	[102, 103]
ATX-POLR3B	HLD8	614381	Japan, Caucasians, Syria, African American, Mediterranean	Intellectual disability, vertical gaze limitation, hypodontia, hypogonadotropic hypogonadism, myopia, mild hyperreflexia, short stature, infantile to childhood onset, diffuse cerebral hypomyelination with partly myelinated internal capsule, cerebellar atrophy, thin corpus callosum, T2 hypointense thalamus	[104, 105]
ATX-RNF216	Ataxia and hypogonadotropism/GHS	212840	Middle East, Caucasians	Hypogonadotropic hypogonadism, dementia, occasional chorea, childhood to young adult onset, cerebellar atrophy, cerebral white matter anomalies	[106, 107]
SCYL1	SCAR21	616719	European, Middle East, Cuba, Ashkenazi Jews	Transient episodes of liver failure, intention tremor, peripheral sensorimotor neuropathy, mild cognitive impairment, occasional short stature, infantile to childhood onset, cerebellar vermis atrophy	[108, 109]
ATX-SNX14	SCAR20	616354	Portugal, Middle East, North Africa, Central Asia	Intellectual disability, developmental delay, macrocephaly, dysmorphism, hypotonia, skeletal anomalies, occasional sensorineural hearing loss, infantile onset, cerebellar atrophy	[110, 111]
SLC9A1	LIKNS/SCAR19	616291	Turkey, Han Chinese	Occasional sensorineural hearing loss, mild psychomotor delay, infantile to childhood onset, progressive cerebellar atrophy	[112, 113]
ATX-SPTBN2	SCAR14/SPARCA1	615386	Middle East, Egypt, North America	Cognitive impairment, developmental delay, nystagmus, hypotonia, occasional tremor, infantile to childhood onset, cerebellar atrophy; allelic to SCA5	[114, 115]
ATX-STUB1	SCAR16	615768	China, Middle East, Caucasians	Pyramidal signs, variable cognitive impairment, occasional hypogonadism, variable age at onset, cerebellar atrophy; allelic to SCA48	[116, 117]
TDP2	SCAR23	616949	Ireland, USA	Seizures, developmental delay, dysmorphism, hypotonia, hypersomnia, failure to thrive, infantile to childhood onset, absence of cerebellar atrophy	[118, 119]
ATX-TPP1	SCAR7	609270	The Netherlands, African American population	Occasional pyramidal signs, posterior column involvement, tremor, square-wave jerks, nystagmus, childhood to adolescent onset, pontocerebellar atrophy; allelic to CLN2	[120, 121]
HSP/ATX-UCHL1	SPG79	615491	Norway, Turkey	Optic atrophy, nystagmus, intention tremor, pyramidal signs, dorsal column involvement, mild cognitive impairment, childhood onset, cerebellar atrophy	[122, 123]
ATX-VLDLR	CAMRQ1/DES	224050	North American Hutterite population, Middle East, Europe	Nonprogressive cerebellar ataxia, moderate to severe intellectual disability, hypotonia, strabismus, delayed ambulation with occasional quadripedal gait, seizures, congenital to infantile onset, inferior cerebellar hypoplasia, pontine hypoplasia, cortical gyral simplification	[124, 125]
VPS13D	SCAR4	607317	Europe, USA, French Canadian, Egyptian, Javanese	Pyramidal signs, axial hypotonia, oculomotor abnormalities, chorea or dystonia, cognitive impairment, infantile to adult onset, cerebellar atrophy, basal ganglia T2/F hyperintensity	[126, 127]
ATX-WDR81	CAMRQ2/DES2	610185	Turkey, Yemen	Occasional quadrupedal gait, intellectual disability, congenital onset, cerebellar hypoplasia; allelic with Congenital hydrocephalus type 3 with brain anomalies	[128, 129]
XRCC1	SCAR26	617633	India, Pakistan	Oculomotor apraxia with nystagmus, peripheral sensorimotor axonal neuropathy, cognitive impairment, childhood to adult onset, progressive cerebellar atrophy	[130, 131]

In part inspired from [3]

^1^MDS nomenclature: nomenclature proposed by the Movement Disorder Society Task Force on Classification and Nomenclature of Genetic Movement Disorders [4] with a phenotypical prefix followed by the gene name. *ATX* ataxia, *HSP* hereditary spastic paraplegia, *MYC* myoclonus

^2^*AOA* ataxia with oculomotor apraxia, *ARCA* autosomal recessive cerebellar ataxia, *ARSACS* autosomal recessive spastic ataxia of Charlevoix-Saguenay, *AT* ataxia telangiectasia, *ATLD* ataxia telangiectasia-like disorder, *AVED* ataxia with vitamin E deficiency, *BNS* Boucher-Neuhäuser syndrome, *CA* Cayman ataxia, *CAMRQ* cerebellar ataxia mental retardation with or without quadrupedal locomotion, *DCMA* dilated cardiomyopathy with ataxia, *DES* disequilibrium syndrome, *EAOH* early-onset ataxia with oculomotor apraxia and hypoalbuminemia, *FRDA* Friedreich ataxia, *GHS* Gordon Holmes syndrome, *HLD* hypomyelinating leukodystrophy, *IOSCA* infantile onset spinocerebellar ataxia, *LIKNS* Lichtenstein-Knorr syndrome, *MGCA5* 3-methyglutaconic aciduria type 5, *MIRAS* mitochondrial recessive ataxia syndrome, *MC4D* mitochondrial complex 4 deficiency, *MSS* Marinesco-Sjogren syndrome, *MTDPS7* mitochondrial DNA depletion syndrome 7, *NBIA* neurodegeneration with brain iron accumulation, *OMCS* Oliver McFarlane syndrome, *PBD* peroxisome biogenesis disorder, *PEOA3* progressive external ophthalmoplegia with mitochondrial DNA deletions, autosomal dominant 3, *PHARC* polyneuropathy hearing loss ataxia retinitis pigmentosa and cataract, *SANDO* sensory ataxic neuropathy with dysarthria and ophthalmoparesis, *SCAE* spinocerebellar ataxia with epilepsy, *SCAN1* spinocerebellar ataxia with axonal neuropathy 1, *SCAR* spinocerebellar ataxia autosomal recessive, *SeSAME* seizures sensorineural deafness ataxia mental retardation and electrolyte imbalance, *SPAX* spastic ataxia, *SPG* spastic paraplegia, *UMN* upper motor neuron, *ZSD* Zellweger spectrum disorder

**Table 2:** Other metabolic or complex autosomal recessive disorders that have ataxia as an associated feature

MDS nomenclature^1^ or gene name	Alternate nomenclature^2^	OMIM	Additional clinical clues	References
ATX-AHI1 ATX-ARL13B ATX-CEP290 ATX-CC2D2A ATX-OFD1 ATX-TMEM231 ATX-TMEM67 ATX-RPGRIP1L Others	Joubert syndrome (including COACH syndrome)	Many, see 213300	Developmental delay, ataxia, hypotonia, neonatal breathing abnormalities, intellectual disability, nephronophthisis, congenital onset, agenesis of the cerebellar vermis with molar tooth sign; in COACH syndrome, associated with ocular colobomas and hepatic fibrosis	[132, 133]
ATX-ALDH5A1	Succinic semialdehyde dehydrogenase deficiency	603147	Developmental delay, intellectual disability, language dysfunction, hypotonia, hyporeflexia, autistic behavior and hallucinations, infantile to childhood onset, T2 hypersignal in globi pallidi	[134, 135]
ATX-ALG6	CDG1c	603147	Developmental delay, hypotonia, seizures, protein-losing enteropathy, psychiatric manifestations, nystagmus, strabismus, failure to thrive, dysmorphic features, neonatal to infantile onset, occasional brain atrophy	[136, 137]
DYT/ATX-ATP7B	Wilson disease	277900	Tremor, dystonia, parkinsonism, choreoathetosis, liver disease, psychiatric involvement, Kayser-Fleischer rings, childhood to adult onset, T2 hypersignal in basal ganglia or brainstem	[138]
ATP8A2	CAMRQ4	615268	Global development delay, cognitive impairment, microcephaly, ataxia or quadrupedal gait, choreoathetoid movement, congenital onset, cerebellar and cerebral atrophy or delay in myelination	[139, 140]
HSP/ATX-B4GALNT1	SPG26	609195	Pyramidal signs, amyotrophy, progressive hyporeflexia, cognitive impairment, axonal peripheral neuropathy, occasional cerebellar ataxia and extrapyramidal signs, scoliosis, childhood to teenage onset, cerebral cortical atrophy, T2/F white matter hyperintensity	[141]
ATX-BTD	Biotinidase deficiency	253260	Seizures, hypotonia, developmental delay, optic atrophy, sensorineural hearing loss, skin rash, alopecia, hepatosplenomegaly, optic atrophy, exacerbation during infections, infantile to childhood onset, white matter anomalies including delayed demyelination	[142, 143]
MYC-CLN5	CLN	256731	Myoclonic epilepsy, psychomotor retardation or regression, ataxia, visual loss, ataxia, infantile to adult onset, cerebellar and cortical atrophy	[144]
NBIA/DYT/PARK-CP	Aceruloplasminemia	604290	Diabetes, dementia, parkinsonism, dystonia, cerebellar ataxia, retinal degeneration, involuntary movements, anemia, low serum and urinary copper, adult onset, decreased signal intensity in thalamus, basal ganglia and dentate nucleus	[145]
MYC/ATX-CSTB1	Unverricht and Lundborg disease/EPM1	254800	Stimulus-sensitive and action-sensitive myoclonus, tonic-clonic generalized seizures, mild cerebellar ataxia, cognitive impairment, emotional lability, childhood to adolescent onset, normal brain MRI	[146]
EIF2B1, EIF2B2, EIF2B3, EIF2B4, EIF2B5	Vanishing white matter disease	603896	Cerebellar ataxia with spasticity, clinical deterioration following head trauma, febrile illness or surgery, infantile to adult onset, symmetric and diffusely abnormal cerebral white matter that appears isointense to CSF	[147, 148]
MYC/ATX-EPM2A MYC/ATX-NHLRC1	Lafora disease	607566	Myoclonus, generalized tonic-clonic seizures, occipital seizures, headaches, behavioral deterioration, rapidly progressive dementia, cerebellar ataxia, spasticity, adolescent onset, normal initial brain MRI with progressive diffuse atrophy	[149, 150]
ERCC4	Xeroderma pigmentosum/Cockayne syndrome	278760	Photosensitivity, solar lentigine growth retardation, microcephaly, ataxia, chorea, cognitive impairment, adolescent to adult onset, cerebellar and brainstem atrophy	[151, 152]
HSP/ATX/NBIA-FA2H	SPG35/FAHN	612319	Spastic paraparesis, pyramidal signs, dystonia, ataxia, dysarthria, optic atrophy, seizures, cognitive impairment, childhood to adolescent onset, T2 subcortical and periventricular white matter hyperintensity, atrophy of the cerebellum and brainstem	[153]
ATX/HSP-FOLR1	Neurodegeneration due to cerebral folate transport deficiency	613068	Developmental regression, hypotonia, myoclonic, tonic or astatic seizures, cerebellar ataxia, chorea, tremor, autism spectrum disorder, occasional pyramidal signs, infantile onset, delayed myelination in cerebral white matter, cerebellar atrophy	[154, 155]
HSP/ATX-GAN1	Giant axonal neuropathy 1	256850	Peripheral sensorimotor neuropathy, weakness, amyotrophy, areflexia, pes cavus, typical frizzly hair, ataxia, nystagmus, pyramidal signs, seizures, cognitive impairment, childhood onset, cerebellar or cerebral white matter T2 hypersignal	[156, 157]
DYT/PARK-GLB1	GM1 gangliosidosis type II	230600	Developmental regression in childhood with gait disorder and cognitive impairment, dystonia, hepatosplenomegaly, ataxia, skeletal dysplasia, cardiomyopathy, infantile to childhood onset, progressive diffuse brain atrophy	[158, 159]
ATX/HSP-HEXA	Tay-Sachs disease	272800	Infantile form with weakness, motor regression, startle reaction, myoclonic jerks, decreased attentiveness, cherry red spots, dementia, blindness. Juvenile form with ataxia, dysarthria, incoordination; adult form with ALS-like symptomatology	[160, 161]
ATX/HSP-HEXB	Sandhoff disease	268800	Similar to Tay-Sachs with organomegaly	[162]
HSD17B4	Perreault syndrome1, D-bifunctional protein deficiency	233400	Sensorineural hearing loss, ovarian dysfunction, ataxia, dysarthria, dysmetria, hyperreflexia, cognitive impairment, sensory neuropathy, childhood onset, cerebellar atrophy	[163, 164]
HSP-KIAA1840	SPG11	604360	Spasticity, ataxia, cognitive impairment, sensorimotor neuropathy, childhood or teenage onset, thin corpus callosum, signal abnormalities in cervical cord	[165, 166]
MYC/ATX-KCTD7	EPM3/CLN14	611726	Multifocal myoclonic seizures, status myoclonus, motor and language regression, intellectual disability, cerebellar ataxia, infantile onset, diffuse cerebral and cerebellar atrophy, T2 periventricular white matter hyperintensity	[167, 168]
ATX-MAN2B1	Alpha-mannosidosis	248500	Dysmorphism, skeletal abnormalities, visceromegaly, sensorineural hearing loss, immunodeficiency, cognitive impairment, psychosis, ataxia, prenatal to adult onset, cerebellar atrophy, partially empty sella turcica, white matter abnormalities	[169]
HSP/ATX-MLC1	Megalencephalic leukoencephalopathy with subcortical cysts	604004	Macrocephaly, initial radiological-clinical discrepancy, eventual motor regression, ataxia, spasticity, epilepsy, cognitive decline, infantile onset, diffuse supratentorial white matter signal anomalies	[170]
ATX-MSTO1	MMYAT	617619	Myalgia, proximal muscle weakness, psychiatric manifestations, developmental delay, tremor, dysmetria, pigmentary retinopathy, growth retardation, neonatal to childhood onset, cerebellar atrophy	[171, 172]
MTTP	Abetalipoproteinemia	200100	Fat malabsorption symptoms, hypocholesterolemia, hypotriglyceridemia, acanthocytosis, sensory loss, hyporeflexia, ataxia, neonatal onset, absence of cerebellar atrophy	[173]
MYC/ATX-NEU1	Neuraminidase deficiency or sialidosis type I and II	256550	Myoclonic epilepsy, visual impairment, cherry red spots, ataxia, hyperreflexia, severe phenotype with dysmorphic features, dysostosis multiplex, hepatomegaly, developmental delay, increased urinary bound sialic acid, variable age at onset, diffuse cerebellar and cerebral atrophy	[174, 175]
NKX6-2	SPAX8 with hypomyelinating leukodystrophy	617560	Nystagmus, developmental delay, hypotonia followed by rapidly progressive spasticity, weakness, dystonia, dysphagia, ataxia, visual impairment, infantile to childhood onset, brain hypomyelination, occasional cerebellar atrophy	[176, 177]
ATX-NPC1 ATX-NPC2	Niemann-Pick type C	257220 607625	Vertical supranuclear ophthalmoplegia, gelastic cataplexy, premature cognitive decline, dystonia, hepatosplenomegaly, respiratory failure, seizures, psychiatric features, neonatal to adult onset, variable cerebellar or cerebral atrophy	[178–180]
OPA1	Behr syndrome	210000	Optic atrophy, pyramidal signs, sensorimotor peripheral neuropathy, cerebellar ataxia, developmental delay, gastrointestinal symptoms, infantile or childhood onset, cerebellar atrophy; allelic to dominant optic atrophy 1	[181, 182]
PEX2	PBD5B/Zellweger spectrum disorder	614867	Hypotonia, seizures, inability to feed, ataxia, hyporeflexia, slow saccades, sensorimotor neuropathy, childhood to adult onset, cerebellar atrophy	[183, 184]
ATX-PEX7	PBD9B	614879	Retinitis pigmentosa, polyneuropathy, ataxia, anosmia, pes cavus, skeletal abnormalities, ichthyosis, hearing loss, cataracts, cardiomyopathy, elevated phytanic acid, childhood or teenage onset, absence of cerebellar atrophy	[185]
ATX-PHYH	Refsum disease	266500	Retinitis pigmentosa, polyneuropathy, increased CSF protein, anosmia, sensorineural hearing loss, ichthyosis, ataxia, teenage onset, elevated serum phytanic acid, absence of cerebellar atrophy	[186]
NBIA/DYT/PARK-PLA2G6	NBIA 2A	256600	Psychomotor retardation or regression, hypotonia followed by spastic quadriparesis, ataxia, strabismus, nystagmus, infantile to teenage onset, cerebellar atrophy and variable iron accumulation in globi pallidi with associated T2 hypointensity	[187, 188]
ATX-PMM2	CDG 1a	212065	Intellectual disability, axial hypotonia, visceral involvement with feeding difficulties and cardiac involvement, dysmorphic features, cerebellar ataxia, strabismus, peripheral neuropathy, retinitis pigmentosa, skeletal abnormalities, infantile to adult onset, cerebellar hypoplasia or atrophy	[189, 190]
PxMD/DYT/ATX-PRRT2	Episodic kinesigenic dyskinesia 1	614386	Seizures, paroxysmal nonkinesigenic dyskinesia, paroxysmal vertigo, episodic ataxia, hemiplegic migraine, rare progressive ataxia, infantile to childhood onset, occasional cerebellar atrophy	[191, 192]
ATX-PTRH2	IMNEPD	616263	Developmental delay, intellectual disability, hypotonia, muscular weakness, demyelinating sensorimotor neuropathy, dysmorphism, ataxia, microcephaly, growth retardation, sensorineural deafness, pancreatic insufficiency, infantile onset, variable cerebellar atrophy	[193, 194]
SEPSECS	PCH 2D	613811	Developmental delay, intellectual disability, hypotonia, nystagmus, microcephaly, seizure, ataxia, spasticity, chorea, congenital to infantile onset, cerebellar and cerebral atrophy, thinning of corpus callosum	[195, 196]
ATX-SLC17A5	Sialic acid storage diseases	604369 269920	Severe neonatal phenotype with ascites, failure to thrive and early death. Milder infantile phenotype with hypotonia, cerebellar ataxia and intellectual disability, infantile to adult onset, hypomyelination, cerebellar atrophy	[197–199]
SLC2A1	GLUT1 deficiency	606777	Epileptic encephalopathy, psychomotor retardation, hypotonia, dystonia, microcephaly, ataxia, spasticity, seizures, infantile onset, absence of cerebellar atrophy	[200, 201]
ATX-SLC52A2	SCAR3/BVVLS2	271250 614707	Sensorimotor neuropathy, optic atrophy, blindness, sensorineural hearing loss, respiratory insufficiency, bulbar involvement, childhood onset, absence of cerebellar atrophy; ataxia is on a spectrum between Brown-Vialetto-Van Laere syndrome type 2 and SCAR3	[202–204]
SLC6A19	Hartnup disorder	234500	Transient manifestations of pellagra, cerebellar ataxia, psychosis, nystagmus and ophthalmoparesis, cognitive impairment, amino aciduria, early onset	[205]
SLC25A46	CMT6B	616505	Optic atrophy, blindness, severe sensorimotor neuropathy, hyporeflexia, amyotrophy, pes cavus, sensory loss in lower limbs, sensitive and cerebellar ataxia, nystagmus, divergent strabismus, neonatal to childhood onset, cerebellar and brain atrophy, T2 hyperintensity in cerebellar white matter	[206, 207]
ATX-SRD5A3	CDG 1q	612379	Hypotonia, intellectual disability, optic nerve atrophy, nystagmus, ocular colobomas, ichthyosis, palmoplantar keratodermia, mild ataxia, congenital to childhood onset, cerebellar vermis hypoplasia	[208, 209]
ATX-TTC19	MC3DN2	615157	Muscular hypotonia progressing to spasticity, developmental delay, neurological regression with loss of language and ambulation, cognitive regression, rapid evolution, axonal motor neuropathy, psychiatric features, infantile to adult onset, cerebral and cerebellar atrophy, T2 hypersignal in basal ganglia, bilateral inferior olive involvement	[210–212]
ATX-WDR73	GMS/SCAR5	251300	Intellectual disability, nephrotic syndrome, microcephaly, hypotonia, epilepsy, optic atrophy, skin abnormalities, infantile to childhood onset, cerebellar and cerebral atrophy	[213, 214]
WFS1	Wolfram syndrome	222300	Diabetes mellitus, optic atrophy, diabetes insipidus, deafness, renal abnormalities, ataxia, intellectual disability, psychiatric features, childhood to adolescent onset, generalized brain and cerebellar atrophy	[215]
WWOX	SCAR12	614322	Tonic-clonic epilepsy, intellectual disability, spasticity, neonatal to childhood onset, variable cerebellar or cerebral atrophy, phenotypic spectrum with infantile epileptic encephalopathy associated with psychomotor retardation and growth retardation	[216, 217]

^1^MDS nomenclature: nomenclature proposed by the Movement Disorder Society Task Force on Classification and Nomenclature of Genetic Movement Disorders [4] with a phenotypical prefix followed by the gene name. *ATX* ataxia, *DYT* dystonia, *HSP* hereditary spastic paraplegia, *MYC* myoclonus, *NBIA* neurodegeneration with brain iron accumulation, *PARK* Parkinsonism

^2^*ALS* amyotrophic lateral sclerosis, *BVVLS2* Brown-Vialetto-Van Laere syndrome type 2, *CAMRQ* cerebellar ataxia mental retardation with or without quadrupedal locomotion, *CDG* congenital disorder of glycosylation, *CLN* neuronal ceroid lipofuscinosis, *CMT* Charcot-Marie-Tooth, *COACH* cerebellar vermis hypoplasia, oligophrenia, congenital ataxia, ocular coloboma, and hepatic fibrosis, *EPM* progressive myoclonic epilepsy, *FAHN* fatty acid hydroxylase-associated neurodegeneration, *GMS* Galloway-Mowat syndrome, *IMNEPD* infantile-onset multisystem neurologic, endocrine, and pancreatic disease, *MC3DN2* mitochondrial complex III deficiency, nuclear type 2, *MMYAT* mitochondrial myopathy and ataxia, *NBIA* neurodegeneration with brain iron accumulation, *PBD* peroxisome biogenesis disorder, *PCH* pontocerebellar hypoplasia, *SCAR* spinocerebellar ataxia autosomal recessive, *SPAX* spastic ataxia, *SPG* spastic paraplegia

### Clinical Approach to a Patient Presenting with Ataxia

**Fig. 1:**
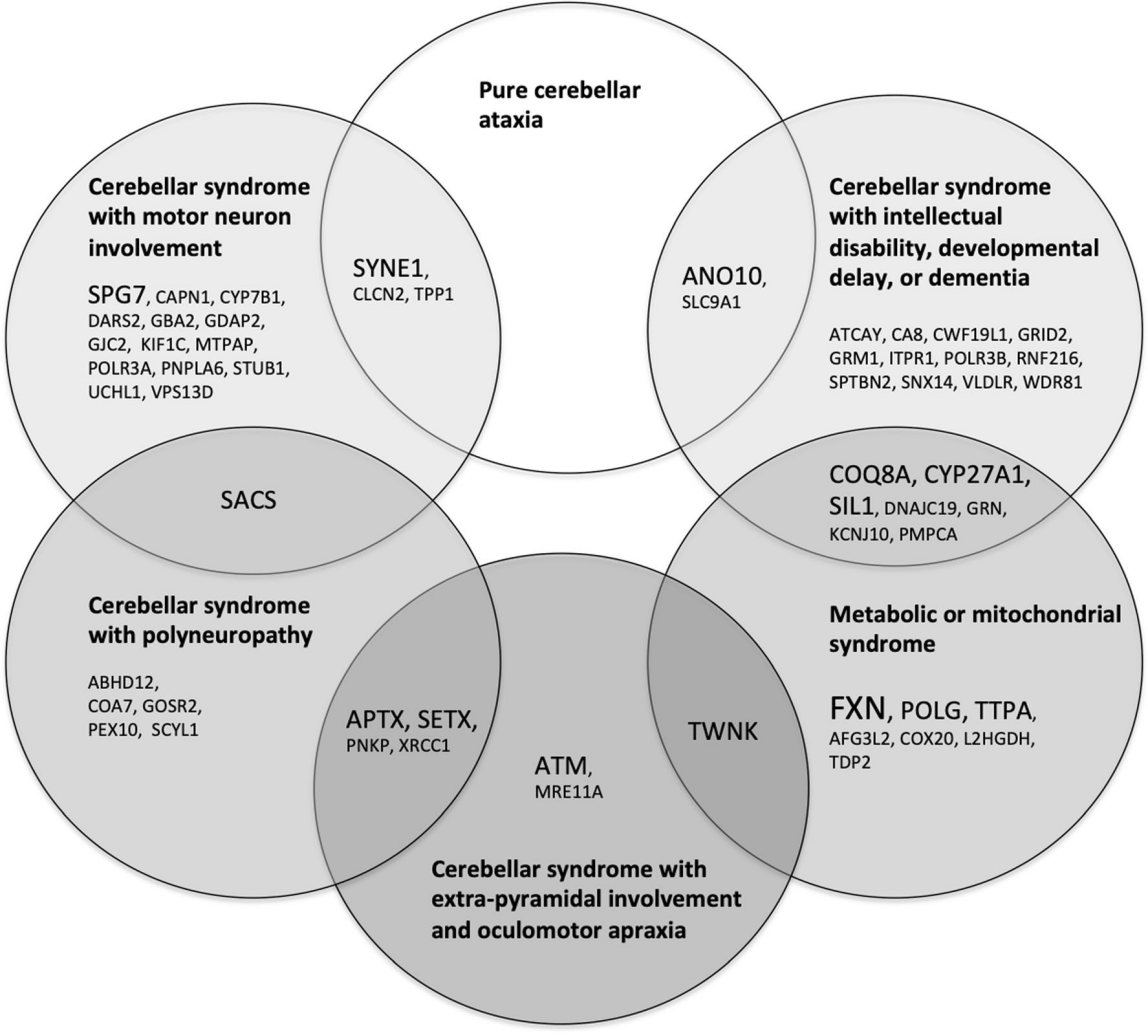
Clinical classification of autosomal recessive ataxias. The gene associated with each primary recessive ataxia is classified according to the most frequent clinical syndrome described for this disorder. Note that some disorders have more complex or variable phenotypes and are placed in the overlapping areas between two categories. Genes presented in larger font represent the most prevalent ataxias

**Fig. 2:**
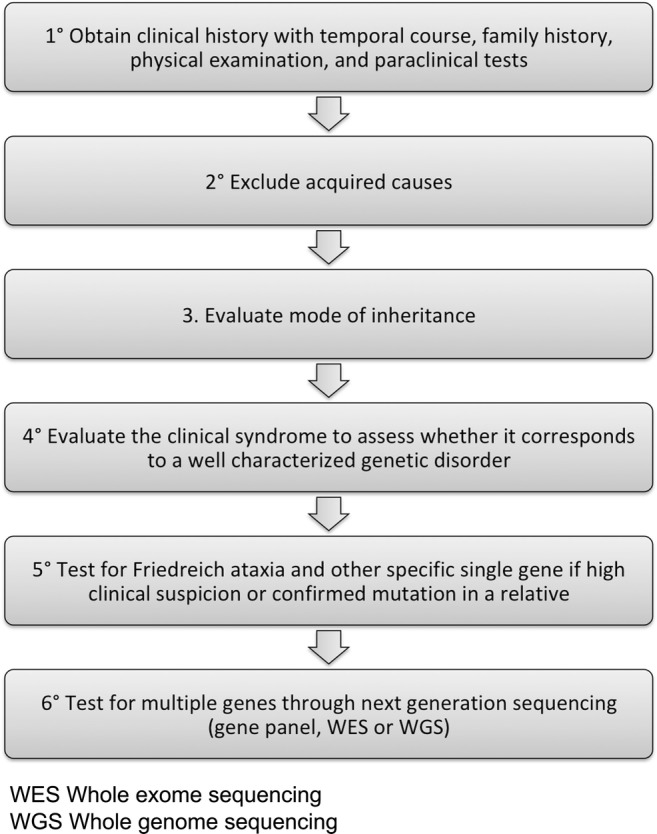
Graphical summary of the clinical approach to a patient presenting with ataxia

1. The first step in evaluating a patient with ataxia is to perform a detailed clinical evaluation that includes a clinical history, a family history, a targeted neurological and systemic physical evaluation, and relevant paraclinical tests. The temporal course is a central element in determining the underlying etiology. Indeed, a chronic progressive evolution over months to years, without trauma or toxin exposure, is suggestive of a hereditary disorder, whereas acute or subacute onset points towards an acquired etiology. A clinical history and physical examination are essential to assess the severity of the cerebellar syndrome and the presence of associated neurological features or systemic involvement. Headache, fever, or an associated autoimmune disorder should prompt the consideration of acquired etiologies. A detailed family history should be obtained to search for relatives with similar symptomatology. Laboratory tests may be useful to rule out acquired causes or as biomarkers for certain disorders. Neuroimaging, preferably with magnetic resonance imaging, is an essential tool to evaluate the presence of cerebellar atrophy or signal anomalies, to search for associated pontine atrophy, and to rule out space-occupying lesions. Electromyography and nerve conduction studies can prove the presence of clinically suspected or subclinical neuropathy and provide evidence of associated myopathy.

2. Following the clinical assessment, one should verify that acquired and treatable causes for ataxia have been excluded. These include vascular disease, trauma, infection, primary or metastatic tumor, excess alcohol consumption, vitamin deficiency, Creutzfeldt-Jakob disease, and immune-mediated cerebellar ataxias such as multiple sclerosis, gluten ataxia, anti-GAD (glutamic acid decarboxylase) ataxia, and paraneoplastic cerebellar degenerations. Clinical evaluation should reveal previous exposure to toxins or traumatic injuries, along with specific signs and symptoms suggestive of infectious, vascular, or metastatic disease. Laboratory tests are useful to identify vitamin deficiencies or autoimmune conditions. Specifically, testing for antibodies involved in paraneoplastic or autoimmune cerebellar degeneration may be particularly useful for patients with a subacute progression, older age at onset, and absence of family history. The paraneoplastic antibodies most associated with cerebellar degeneration are anti-Yo, anti-Hu, anti-Tr, and anti-mGluR1 antibodies; the tumors most often involved are breast and gynecological tumors, Hodgkin lymphoma, and small-cell lung carcinoma [218]. Large paraneoplastic autoantibody panels are now available and may reduce the delay associated with serial testing.

3. Once acquired causes have been ruled out, a genetic etiology may be considered, especially in the presence of a positive family history, early onset, chronic progressive course, or with a set of clinical signs and symptoms that is reminiscent of a well-described genetic disorder. One should bear in mind that a negative family history does not rule out a genetic cause, and sporadic cases may be due to recessive or mitochondrial inheritance, de novo mutations, genetic anticipation, incomplete penetrance, variability in disease expression, paternity error, gonadic mosaicism, or incomplete phenotyping of family members. Indeed, recessive disorders may appear as sporadic in small kindred or with incomplete family history. In other cases, a complete family history should allow identification of the mode of transmission.

4. If autosomal recessive inheritance is suspected, the next step in clinical evaluation is to consider age at onset and clinical signs and symptoms to evaluate if the clinical picture is reminiscent of a well-described disorder. Presentation in infancy suggests ataxia telangiectasia or autosomal recessive ataxia of Charlevoix-Saguenay. Childhood or teenage onset should raise the suspicion for Friedreich ataxia, ataxia with oculomotor apraxia 1 and 2, and POLG-related disorders. Finally, recessive ataxia with onset in adulthood is evocative of autosomal recessive cerebellar ataxia 1 and 3 and spastic paraplegia 7. However, there are large variations in the age at onset of most of the presented disorders, and Friedreich ataxia is one of the best examples with some patients presenting with late-onset (> 25 years of age) or very-late-onset Friedreich ataxia (> 40 years of age). Clinical signs and symptoms may provide clues to identify the mutated gene. Indeed, certain discriminating clinical features or combinations of neurological symptoms may be helpful to guide the clinician towards specific genes (Fig. 1 and Table 1). As one may observe in Fig. 1, none of the autosomal recessive ataxias reported up to now presents with a pure cerebellar phenotype. Even SYNE1-related autosomal recessive cerebellar ataxia 1, which used to be the prototype of a pure cerebellar phenotype [21], has recently been reported to be associated with upper and/or lower motor neuron involvement in 58% of cases, with some rare patients presenting with a very severe early-onset neuromuscular phenotype [22]. The presence of motor neuron involvement, polyneuropathy, extrapyramidal movement disorders, eye movement abnormalities such as oculomotor apraxia, intellectual impairment, and associated multisystemic involvement may guide the clinician towards a particular diagnosis. Some clinical syndromes are particularly evocative of specific disorders. Multisystemic involvement with sensory loss, muscle weakness, cardiomyopathy, diabetes, optic atrophy, and sensorineuronal hearing loss is characteristic of Friedreich ataxia, which is the prototype of a disorder associated with mitochondrial dysfunction. Other associated disorders present with similar features and occasionally epilepsy, retinal involvement, or ophthalmoplegia, such as POLG-related disorders, autosomal recessive cerebellar ataxia 2, and Marinesco-Sjogren syndrome. Extrapyramidal involvement with oculomotor apraxia, elevated α-fetoprotein, and occasional polyneuropathy are typical findings of ataxia telangiectasia, ataxia telangiectasia-like disorder, spinocerebellar ataxia recessive 26, and ataxia with oculomotor apraxia types 1, 2, and 4. Nevertheless, autosomal recessive ataxias are characterized by important phenotypic variability and significant clinical overlap between different pathologies, such that predicting the mutated gene according to the clinical phenotype is prone to errors even for ataxia experts [219]. Some laboratory tests may serve as useful biomarkers for recessive ataxias. Altered levels of vitamin E, α-fetoprotein, albumin, coenzyme Q10, cholesterol, cholestanol, lactate, sex hormones, and gonadotropins have been associated with specific disorders (see Table 1). Dosing of immunoglobulins, very long chain fatty acids, and hexosaminidase may be relevant according to clinical suspicion.

5. Once the clinical assessment is complete, genetic testing is indicated to confirm the mutated gene or allow a more specific diagnosis if the clinical picture is nonspecific. Initial testing should include searching for the Friedreich ataxia-associated trinucleotide repeat expansion in the FXN gene considering the high prevalence of this mutation, its incomplete coverage through the next-generation sequencing methods [1], and the heterogeneous clinical phenotype. Searching for a FXN repeat expansion can be done with frataxin protein analysis or gene analysis with Southern blot or PCR. Moreover, clinicians may consider testing for another specific gene through Sanger sequencing or multiplex ligation-dependent probe amplification (MLPA) if the clinical and paraclinical data are highly evocative of a particular disorder, if there is a confirmed mutation in a relative or in isolated populations where selected disorders are highly prevalent. Finally, a panel for the dominantly inherited CAG-repeat expansion spinocerebellar ataxias may also be considered as part of the initial assessment if family history is inconclusive regarding the mode of inheritance and considering the high prevalence of these mutations and their incomplete coverage through the next-generation sequencing methods [1].

6. If single gene testing does not provide a molecular diagnosis, one should consider the high-throughput NGS methods either with a multigene panel, whole exome sequencing, or whole genome sequencing. Several studies have demonstrated the efficacy and cost efficiency of multigene panels [220], targeted exome sequencing [219, 221], or whole exome sequencing [222, 223], with a diagnostic yield varying between 18 and 80%. The highest yield is obtained for patients with early-onset ataxia and positive family history and consanguinity among parents. NGS panels allow for better coverage of included genes and reduce the volume of genetic variants that are unrelated to the clinical phenotype, while exome sequencing may reveal mutations in genes that were not previously known to be associated with ataxia [1]. Whole genome sequencing may be considered in selected cases with appropriate genetic counseling, but its diagnostic yield is uncertain [224]. Once genetic testing is completed and a pathogenic mutation has been identified, it is of primary importance to provide specialized genetic counseling for the patient and his or her relatives along with symptom management and disease treatment when available. Figure 2 presents a graphical summary of the proposed clinical approach.

### Pathophysiological Mechanisms Underlying Autosomal Recessive Cerebellar Ataxias

**Fig. 3:**
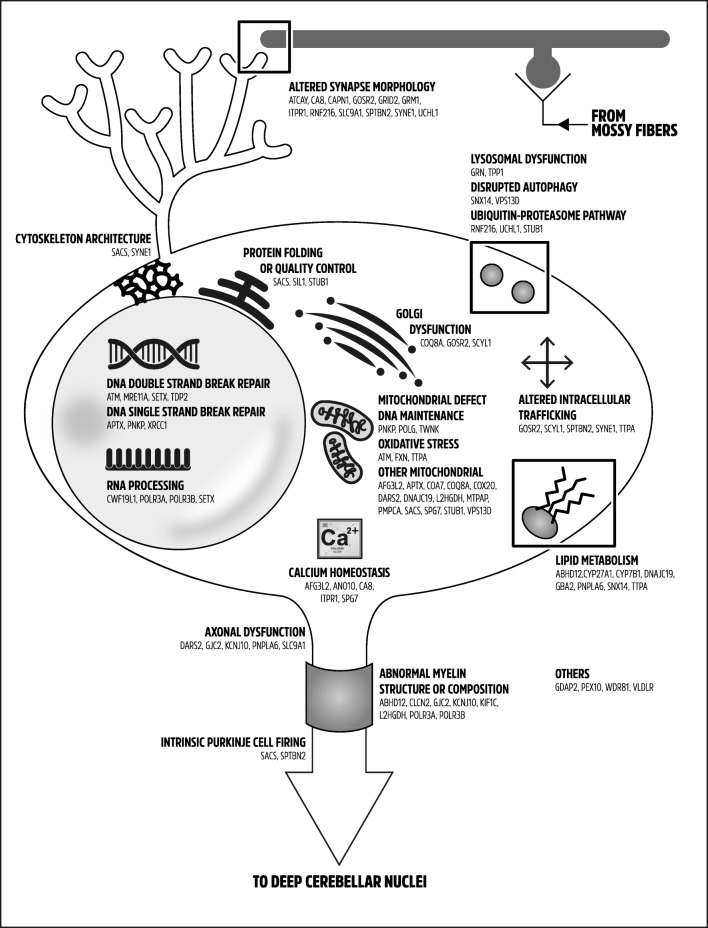
Pathophysiological classification of autosomal recessive ataxias. A Purkinje cell is depicted along with a granule cell and parallel fibers. Subcellular organelles and structures are represented graphically. Each gene is classified at one or more subcellular localizations according to the different metabolic pathways involved

The importance of a proper recessive ataxia classification goes beyond the clinical diagnosis perspective. Autosomal recessive ataxias can be regrouped according to the deficient cellular and metabolic pathways involved, which provide a better understanding of cerebellar physiology and of its selective vulnerability to certain metabolic defects. This is also essential from a therapeutic perspective, as disorders that belong to the same metabolic pathway may respond to the same treatment options, indicating potential for drug repurposing. Figure 3 presents a pathophysiological classification of autosomal recessive ataxias. Certain genes are presented more than once since some proteins are involved in several metabolic pathways or may interfere with other cellular processes as they accumulate in neurons or glial cells. Table 3 presents a more detailed listing of the pathogenic pathways involved along with relevant references.

**Table 3:** Detailed pathogenic mechanisms involved in autosomal recessive cerebellar ataxias

Pathophysiological mechanism		Genes involved	References
Mitochondrial defect	Mitochondrial DNA maintenance	PNKP, POLG, TWNK	[225–227]
	Mitochondrial protein synthesis or quality control	AFG3L2, PMPCA, SPG7	[94, 228, 229]
	Increased reactive oxygen species and oxidative stress	ATM, FXN, TTPA	[230–233]
	Coenzyme Q10 metabolism	APTX, COQ8A	[234, 235]
	Altered mitochondrial dynamics	SACS, VPS13D	[127, 236–238]
	Mitochondrial respiratory chain assembly	COA7, COX20	[54, 55]
	Mitochondrial RNA maturation and processing	DARS2, MTPAP	[91, 239, 240]
	Others	DNAJC19, L2HGDH, STUB1	[241–243]
DNA break repair dysfunction	Double-strand break repair	ATM, MRE11A, SETX, TDP2	[118, 230, 244, 245]
	Single-strand break repair	APTX, PNKP, XRCC1	[130, 225]
RNA transcription or processing defect		CWF19L1, POLR3A, POLR3B, SETX	[57, 104, 246]
Synaptic dysfunction	Aberrant morphology at the PC/parallel fibers synapse	CA8, CAPN1, GRID2, ITPR1	[50, 247–249]
	Impaired dendritic architecture	SPTBN2, SYNE1 (MF/CGN synapse)	[250, 251]
	Dysregulation of glutamate transmission	ATCAY, GRM1	[252, 253]
	Others	GOSR2, RNF216, SLC9A1, UCHL1	[254–257]
Abnormal cytoskeleton architecture		SACS, SYNE1	[251, 258]
Abnormal protein folding or quality control		SACS, SIL1, STUB1	[259–261]
Golgi apparatus dysfunction		COQ8A, GOSR2, SCYL1	[235, 255, 262]
Calcium homeostasis dysregulation		AFG3L2, ANO10, CA8, ITPR1, SPG7	[79, 247, 263–266]
Lysosomal dysfunction		GRN, TPP1	[267, 268]
Disrupted autophagy		SNX14, VPS13D	[238, 269]
Defective ubiquitin-proteasome pathway		RNF216, STUB1, UCHL1	[256, 257, 261]
Altered intracellular trafficking		GOSR2, SCYL1, SPTBN2, SYNE1, TTPA	[250, 251, 255, 262, 270]
Altered lipid metabolism		ABHD12, CYP27A1, CYP7B1, DNAJC19, GBA2, PNPLA6, SNX14, TTPA	[60, 233, 241, 269, 271–274]
Axonal dysfunction		DARS2, GJC2, KCNJ10, PNPLA6, SLC9A1	[239, 274–277]
Abnormal myelin structure or composition		ABHD12, CLCN2, GJC2, KCNJ10, KIF1C, L2HGDH, POLR3A, POLR3B	[242, 276–281]
Disrupted intrinsic Purkinje cell firing		SACS, SPTBN2	[250, 282]
Abnormal cellular stress response		GDAP2	[68]
Peroxisome dysfunction		PEX10	[92]
Impaired mitosis		WDR81	[283]
Abnormal neuronal migration		VLDLR	[284]

*CGN* cerebellar granule neuron, *MF* mossy fiber, *PC* Purkinje cell

Certain pathways are predominantly involved, notably mitochondrial dysfunction, which may result from abnormal mitochondrial DNA maintenance with progressive mutagenesis, defective mitochondrial protein synthesis and quality control, increased levels of reactive oxygen species and oxidative stress, deficient coenzyme Q10 metabolism, altered mitochondrial dynamics, defective mitochondrial chain assembly, or abnormal mitochondrial RNA maturation and processing (Table 3). Interestingly, many of the disorders caused by mitochondrial dysfunction also present with a mitochondrial clinical syndrome as shown in Fig. 1. Disorders of DNA repair mechanisms are also common, with double-strand break repair pathway or single-strand break repair complexes predominantly involved. Pathogenic mutations in these genes are also associated with a susceptibility to ionizing radiations and predisposition for cancers, but the neurological syndrome is characterized by cerebellar involvement and extrapyramidal movement disorders. It remains debated whether defective DNA repair is the main pathogenic mechanism causing the neurological phenotype [230], but the fact that several interacting genes in this pathway are involved in degenerative cerebellar ataxias suggests that the cerebellum has a peculiar susceptibility to DNA damage for which the underlying mechanism is not understood. Finally, altered synaptic morphology or synaptic dysfunction of Purkinje cells (PC) is frequently involved in recessive ataxias and is associated with aberrant morphology at the PC/parallel fiber synapse, impaired dendritic architecture, or dysregulation of glutamate transmission. Other disorders have been implicated in synaptic dysfunction through indirect evidence, for example, SLC9A1, which localizes in presynaptic terminals and is involved in the modulation of synaptic activity [254, 275]. Of interest, many of these disorders are characterized by significant cognitive impairment that goes beyond what is expected in the cerebellar cognitive-affective syndrome and cause intellectual disability, developmental delay, or dementia, highlighting the importance of synaptogenesis in cognitive development.

## Discussion

We present a new clinical classification of autosomal recessive ataxias in parallel with a pathophysiological classification. The objective of this classification is to provide a tool for clinicians and researchers that facilitates the understanding of this complex group of disorders and defines this field of research. This work is based on the results of our systematic scoping review of the literature [3]. We updated this literature review and regrouped a panel of 12 international ataxia experts to build a consensus on the definition and classification of cerebellar ataxias. The task force vision is that a classification goes beyond the listing of disorders and must organize diseases in a way that allows better understanding and clinical mastery of this group of disorders. Hence, we proposed a clinical classification along with a pathophysiological classification, which enabled us to observe that there is significant overlap between these two classifications, highlighting how clinical presentation is in some cases a good projection of the underlying biochemical defect. This has potential applications from bench to bedside since treatments that address a specific pathogenic pathway may have therapeutic potential in all disorders in which this pathway is affected. The clinical classification is presented along with a structured clinical approach to a patient presenting with ataxia, which is intended as a clinical tool for expert and nonexpert clinicians. Despite the increasing accessibility of the NGS techniques, there remains a critical place for clinical judgment in the prescription of genetic tests and interpretation of results, taking into account the technical limitations and risk of finding variants of unknown significance. Recently, Renaud and colleagues published the results of a diagnostic algorithm for recessive ataxias that integrates 124 clinical features to propose three potential diagnoses among a list of 67 recessive disorders that may present with ataxia [285]. This is a very promising tool, but its pragmatic impact on molecular testing strategy, final diagnostic rate, patient management, or time efficiency remains to be validated. In the meantime, it is essential for clinicians to be at ease with a general approach to recessive ataxias with the NGS techniques often permitting molecular diagnosis when the clinical picture is nonspecific.

One of the major strengths of this classification proposal is that it is based on a consensus from a panel of international ataxia experts, thereby ensuring a proper representation of regional differences in the prevalence and clinical approach to ataxias. Moreover, the literature search was based on a systematic scoping review of the literature whose methodology has been published before and which permitted an unbiased appraisal of all potentially relevant articles. Nevertheless, there are some limitations to this classification proposal that are inherent to classifying a group of diseases that evolves very rapidly and that is highly heterogeneous. First, as new evidence emerges regarding the identification of novel ataxia-associated genes and as new phenotypes are described for previously described disorders, this classification will need to be updated. This was highlighted by the significant additions to the list of primary recessive ataxias since the original systematic review was conducted in 2016. Indeed, many new genes and new phenotypes of previously described genes have been reported in only 2 years, which suggests that there is a need for periodic updates to the present classification or an online resource. Moreover, several decisions were made in the elaboration of this classification regarding general orientation, purpose of a classification, inclusion of specific disorders, and classification categories. The lists presented here offer in our opinion the best compromise between synthesis and exhaustiveness for the expert and nonexpert clinician.

Compared with a previously published report by the Movement Disorders Society Task Force [4], we decided to exclude disorders in which cerebellar involvement is a minor or late finding in a complex multisystem phenotype or disorders that are already classified on their own, such as genes associated with Joubert syndrome. The objective was to identify the core disorders that are involved in autosomal recessive ataxias in order to define this field of research and build a classification that would be accessible for all clinicians. Indeed, with the progressive advent of affordable NGS diagnostic testing, we believe that it is most important for clinicians to be at ease with one classification and familiar with the most frequent disorders in their unique ethnical and clinical context. Disorders in which ataxia has been reported as a rare or late finding should be included in large NGS testing strategies, but in our opinion should not be categorized as primary ataxias per se. From this perspective, our classification complements the proposal by the Movement Disorders Society Task Force.

There remain some important challenges to be addressed in the field of autosomal recessive ataxias. First, the issue of a proper nomenclature system has been much debated. Recently, the Movement Disorders Society Task Force proposed a revised naming system based on an ataxia prefix associated with the gene name [4]; this was part of a larger effort to revise the nomenclature of all genetic movement disorders. This system overcomes the limitations of the numbered nomenclature, notably unconfirmed genes, and erroneously attributed phenotypes, but its ease of use by nonexperts and patients remains uncertain. Moreover, some disorders were assigned as many as three phenotypic prefixes while some other disorders that are among the most prevalent causes of recessive ataxia, such as POLG, were not assigned an ataxia prefix. Hence, there remains a debate concerning the attribution of prefixes and the integration of this naming system with other fields in neurology and other specialties as many genes involved in ataxia have very complex multisystem phenotypes. Finally, one of the most important challenges in this field of orphan diseases is to develop targeted treatment strategies that address the pathogenic mechanism underlying symptom progression. To this end, we believe that identifying common pathophysiological pathways may provide an opportunity for drug repurposing or enlarge the number of patients that are admissible for drug trials in order to find treatments for these rare but debilitating diseases.

## Conclusion or Summary

We present a clinical and a pathophysiological classification of autosomal recessive cerebellar ataxias along with a clinical approach to a patient presenting with ataxia. This classification is the result of a consensus among a panel of international experts, and it promotes a unified understanding of autosomal recessive cerebellar disorders for clinicians and researchers.
